# Emerging challenges in meeting physiotherapy needs during COVID-19 through telerehabilitation

**DOI:** 10.1186/s43161-020-00018-4

**Published:** 2020-12-10

**Authors:** Joseph Ayotunde Aderonmu

**Affiliations:** Department of Physiotherapy, University College Hospital, Ibadan, Nigeria

**Keywords:** Physiotherapy, COVID-19, Pandemic, Telerehabilitation, Developing countries

## Abstract

**Background:**

Physiotherapy plays a significant role in rehabilitation. However, the emergence of the coronavirus disease 2019 (COVID-19) has posed a big challenge to its practice, especially regarding the level of contact with patients. There is a dire need for the exploration of rehabilitation options, other than in-person contacts, to limit the spread of the virus. This article explores telerehabilitation, its outcomes, and the challenges involved in the continuum of care of patients by physiotherapists in the face of the pandemic.

**Body:**

Telerehabilitation is a vital tool which utilizes technology to link practitioners to patients. With its previous history of favorable outcomes for the rehabilitation of certain conditions, telerehabilitation has been widely recommended. However, issues exist on how well it can bridge the gap of physical touch in physiotherapy, its effectiveness in terms of outcomes and satisfaction across various conditions and large population sizes, and finally, its cost and effects, especially in developing countries.

**Conclusion:**

Telerehabilitation is a necessary adaptation to ensure continued physiotherapy service delivery during the pandemic. However, more quality studies are recommended to evaluate its effectiveness and outcomes. Also, measures to ensure that developing countries are catered for in meeting the rising demands for physiotherapy services using telerehabilitation should be carried out.

## Background

Since the first report of the coronavirus disease 2019 (COVID-19) caused by novel severe acute respiratory syndrome coronavirus-2 (SARS-CoV-2) was made in Wuhan, Hubei Province of China, on December 31, 2019 [1–3], the global community has experienced devastating consequences. This is supported by the fact that the World Health Organization (WHO), in March 2020, declared the COVID-19 outbreaks a pandemic [4]. The development was followed by several national and international orders such as social (physical) distancing and other preventive behavior change measures to control the spread of the virus, such as regular hand washing and use of face masks [5].

Amidst these realities, rehabilitation has been favored to be of extreme importance and has a crucial role to play during the pandemic [4]. This role can be provided to both patients with COVID-19, and even population without the virus [4]. Rehabilitation plays a vital part in improving health for patients with severe symptoms of COVID-19 [6]. It improves health outcomes by promoting health and functioning, working towards prompt discharge from admission facilities, and preventing relapse and deterioration following discharge [7]. For patients with specific conditions without COVID-19, rehabilitation has been recognized as an essential service, whose delay or stoppage can lead to a decline in health outcomes [8, 9].

These rehabilitation roles are carried out by a multidisciplinary team of which physiotherapy forms a vital component [4]. In the face of the COVID-19 pandemic, physiotherapy plays a frontline role in the acute and post-acute rehabilitation phases of COVID-19 patients and the continuation of rehabilitation for people with disabilities and the elderly with appropriate modifications to the delivery of services to ensure safety as the pandemic demands [10]. The essentiality of physiotherapy services despite the pandemic poses several concerns which call for attention and possible challenges for physiotherapy practitioners. For the reason that physiotherapists are frontline workers during the pandemic, they are at an increased risk of exposure for COVID-19 [4]. This fact has resulted in several measures such as interruption of face-to-face consultations [11], increased adherence to infection, prevention, and control guidelines and use of personal protective equipment (PPE) [4, 10]. Other measures adopted are productivity optimization for staff, modification of shifts to ensure round the clock coverage, supporting workforce physical and mental health, and changes in the mode of service delivery [4, 10].

During the pandemic, physiotherapists have explored a viable service delivery option of remote consultations (also known as telehealth or telerehabilitation) [12] to ensure a continuation of delivery of rehabilitation services for patients without COVID-19. This is also to limit long-term consequences of the complete termination of physiotherapy provision that can lead to increased demand for physiotherapy and high level of disability in the future [10]. This means that expectations are placed on physiotherapy despite the limitations and restrictions imposed by the pandemic. Therefore, this review article examines telerehabilitation, its outcomes, and challenges that are associated with the ongoing coronavirus pandemic with possible directions for research towards achieving greater efficiency in physiotherapy service delivery.

## Telerehabilitation during the COVID-19 pandemic

Conventionally, for an individual to access the services of a physiotherapist, there is a need for a scheduled physical appointment to meet with the physiotherapist at a designated location. However, the advent of technology has bridged the gap that might exist between locations, practitioners, and patients [13]. With continually improving communication technology, technology has been employed to provide remote healthcare services [14], and this is the concept of telerehabilitation.

Brennan et al. [15] (2010) described telerehabilitation as habilitation or rehabilitation services that are provided by a rehabilitation professional remotely, which is useful for assessment, monitoring, prevention, intervention, supervision, education, consultation, and counseling. It comprises the use of videoconferencing via the internet, phone calls, and virtual reality systems, where remote interaction with patients can either be real-time or pre-recorded [16].

Various researches have supported and recommended the application of telerehabilitation to the practice of physiotherapy, especially with the rising levels of sophistication of technology being experienced [17, 18]. With the current global pandemic, many physiotherapy services across different countries have had to rely on it [10, 19]. Telerehabilitation has been extremely useful for populations requiring physiotherapy who are not infected with the virus, especially the elderly population whose ages make more vulnerable to the infection, and people with various disabilities or pre-existing health conditions [4].

However, challenges exist in this emerging field of telerehabilitation, which is still being tackled before the COVID-19 pandemic, and are still being faced in the heat of the pandemic:

## Physical contact in physiotherapy

It is not entirely unexpected that there would be concerns about telerehabilitation, mainly as it eliminates the physical contact which usually exists between the physiotherapist and the patient during the sessions of treatment. A study carried out by Roberts and Bucksey [20] (2007) reported touch (54%) of the non-verbal communication between patient and physiotherapist, eye gaze by physiotherapist (32%), and eye gaze by patients (84%) during physiotherapy sessions. Also, many physiotherapists accept touch to be one of the profession’s critical distinguishing skills that has been an integral part of the definition of the physiotherapy practice [21]. Some of the popular purposes of touch include assessment (such as in palpation), provision of intervention (such as in massage), and assistive touch [22].

Thus, it is not out of place to still find in-person physiotherapy an essential tool to provide the touch component of physiotherapy, a feat which telerehabilitation might not be able to achieve. Also, telerehabilitation has been said not to primarily be a replacement for future in-person rehabilitation and is not a readily suitable option for all categories of patients [10]. Therefore, a good query perhaps might be on how technology, through telerehabilitation, will create an avenue for “touch” in a profession where it (touch) can be a fundamental need for both assessments and provision of interventions.

## Effectiveness of telerehabilitation versus in-person physiotherapy: emphasis on outcomes

Outcomes and effectiveness universally drive the health system in service delivery. Therefore, it comes by no surprise that there have been several attempts by concerned parties, especially clinicians, educators, and health managers, to evaluate the effectiveness of telerehabilitation in comparison to in-person physiotherapy.

There have been many attempts in the literature to compare outcomes of in-person physiotherapy care with telerehabilitation, and evidence has been promising for telerehabilitation for specific conditions such as cancer, heart conditions, musculoskeletal disorders, and depression [23, 24]. However, a systematic review which compared the effectiveness of telerehabilitation and in-person rehabilitation on activities of daily living in stroke reported that “there is only low or moderate-level evidence testing whether telerehabilitation is a more effective or similarly effective way to provide rehabilitation [13]”. van Egmond [25] et al. (2017), in a systematic review concluded that the quality of life could be potentially improved through telerehabilitation in surgical patients, yet reported unclear effectiveness on physical outcomes of patients.

Certainly, telerehabilitation holds promising potentials and successes for the rehabilitation of patients during the period of the pandemic. However, more quality studies are still needed to demonstrate the overall effectiveness and the satisfaction of patients and clinicians as well report encountered challenges in telerehabilitation, especially regarding the practice of physiotherapy during the COIVD-19 pandemic.

## Costs and effects

The immense usefulness of telerehabilitation cannot be considered without equally discussing the cost implications of making it work. In developed countries such as the USA, UK, Canada, Netherlands, and Australia, sizeable studies have been carried out on the concept of telerehabilitation [13, 25, 26], which has led to useful findings to what is currently known about the field.

However, the story is not the same in the developing countries where many challenges are still being faced in terms of the provision and accessibility to stable technology. Appropriate devices and gadgets and accessibility to excellent Internet connections both on the part of the clinician and also the patient have served as a substantial limiting factor to the progress of telerehabilitation in these countries. Odole et al. [27] (2015) reported six perceived challenges to telerehabilitation by Nigerian physiotherapists, which were inadequate and underdeveloped infrastructure, the context of ethical issues, training of physiotherapists/patients’ literacy need, physiotherapy-patient’s contact, cultural issues, and financial implications. Similarly in India, there has been a limited report of studies which have examined the efficiency of telerehabilitation [28]. Although, preliminary studies carried out on feasibility and acceptance of telerehabilitation among geriatric clients produced promising outcomes [29].

The importance of this will be seen in the way the COVID-19 pandemic has severely altered the delivery of physiotherapy services to patients who should have been regularly seen and treated in order to maintain their health and wellness in these countries through the opportunities embedded in telerehabilitation. A search in literature for the use of telerehabilitation in developing countries at the time of writing of this article also produced no result. Thus, more considerable efforts need to be put in place to enable these regions benefit from the immense advantages of telerehabilitation, especially at a time where greater access to physiotherapy might be made possible through the medium.

## Conclusion

Expectations from physiotherapy in rehabilitation has not changed despite the COVID-19 pandemic. Telerehabilitation remains widely recommended for the perpetuation of rehabilitation services, especially to patients who are at high risk of contracting and developing severe complications of the virus, and even the population who have to comply with social (physical) distancing measures in place. However, challenges exist on how telerehabilitation can bridge the gap between physical contact needed for certain assessments and interventions by the physiotherapist, clearer outcomes from its use for a wide variety of conditions, and cost implications which might have limited its application across various climes, especially in developing countries. Further studies are therefore necessary to improve the existing telerehabilitation technologies, especially on those that can find easy applicability in developing countries in terms of costs and existing technological infrastructure most especially during the pandemic. Also, quality studies that would evaluate the performance of telerehabilitation in settings where it is being practiced during the pandemic would contribute significantly to what is known already about the field.

## Data Availability

Not applicable

## Abbreviations

COVID-19: Coronavirus disease 2019
SARS-CoV-2: Severe acute respiratory syndrome coronavirus-2
WHO: World Health Organization
